# Supplementary material for: Orthogonal translation enables heterologous ribosome engineering in E. coli
Source: Nat Commun. 2021 Jan 26;12:599. doi: 10.1038/s41467-020-20759-z (PMC7838251; doi:10.1038/s41467-020-20759-z)
Supplement: Supplementary file 1 — Supplementary Information [file 41467_2020_20759_MOESM1_ESM.pdf]

Orthogonal Translation Enables Heterologous Ribosome Engineering in *E. coli*

Natalie S. Kolber[1], Ranan Fattal[1], Sinisa Bratulic[1], Gavriela D. Carver[1] and Ahmed H. Badran[1]

[1] *Broad Institute of Harvard and MIT, Cambridge, MA, 02142*

*Correspondence should be addressed to: Ahmed H. Badran: ahbadran@broadinstitute.org

TABLE OF CONTENTS

**SI Figure 1**	Comparison of SQ strain complementation and orthogonal translation pipelines
**SI Figure 2**	Benchmarking and extending the orthogonal translation reporter system
**SI Figure 3**	A superfolder GFP-derived leader sequence improves the function of orthogonal reporters
**SI Figure 4**	Divergent O-rRNA activities are not improved following intergenic sequence replacement
**SI Figure 5**	R-protein supplementation significantly improves *A. baumannii* O-rRNA function
**SI Figure 6**	Dissection of LSU r-proteins that improve *A. macleodii* O-rRNA activity
**SI Figure 7**	Investigation of the contributions of the identified r-proteins S20, S16, S1, and S15
**SI Figure 8**	*Enterococcus faecalis* 16S rRNA helices with low sequence identity to *E. coli*
**SI Figure 9**	Erythromycin sensitivity of wildtype and A2058U 23S rRNAs in SQ171 cells
**SI Figure 10**	Benchmarking the ERY-dependent orthogonal translation reporter system
**SI Figure 11**	Analysis of heterologous r-protein sequence similarity to *E. coli* homologs
**SI Table 1**	Summary of heterologous and orthogonal ribosome translation data
**SI Table 2**	Tabulated erythromycin IC_{50} values for wildtype and 23S rRNA A2058U rRNAs
**SI Table 3**	Summary of stapled heterologous ribosome experiments
**SI Table 4**	Primers used in this study
**SI Table 5**	Reporter plasmids used in this study
**SI Table 6**	Species names and GTDB representative genomes used to construct phylogenetic tree
**SI Table 7**	Names of rRNA and r-protein expression plasmids used in this study
**SI Table 8**	Addgene IDs of deposited plasmids

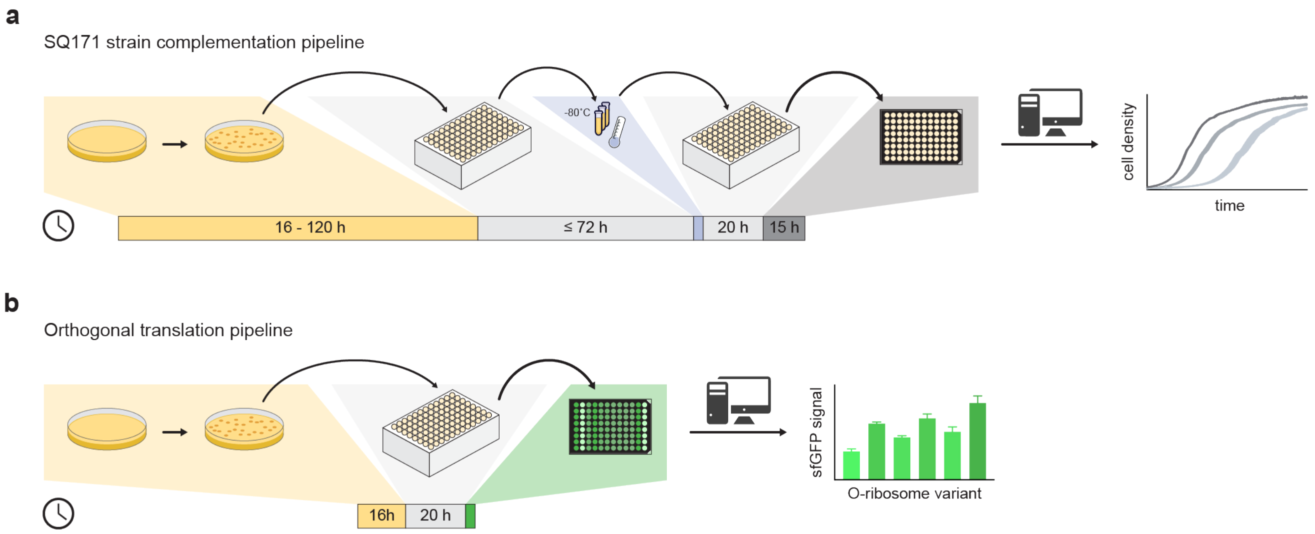

Supplementary Figure 1 | Comparison of SQ strain complementation and orthogonal translation pipelines. a) To evaluate heterologous rRNAs (ribosomal RNAs) via SQ strain complementation, rRNA plasmids are transformed into the SQ171 strain after which it may take up to 120 hours to observe colonies. Colonies are grown up in liquid medium +/- kanamycin to evaluate for pSacB persistence, which may take up to 3 days, after which cultures are glycerol stocked. Finally, stocked strains are revived, grown overnight in liquid medium and back-diluted to initiate a growth curve. b) To evaluate heterologous rRNAs via orthogonal translation, O-rRNA plasmids are transformed alongside the reporter plasmid and plates are incubated overnight. Colonies are then picked into media and grown overnight, after which sfGFP (superfolder GFP) fluorescence is quantified. Detailed experimental conditions for both assays are described in the Methods section. h = hours.

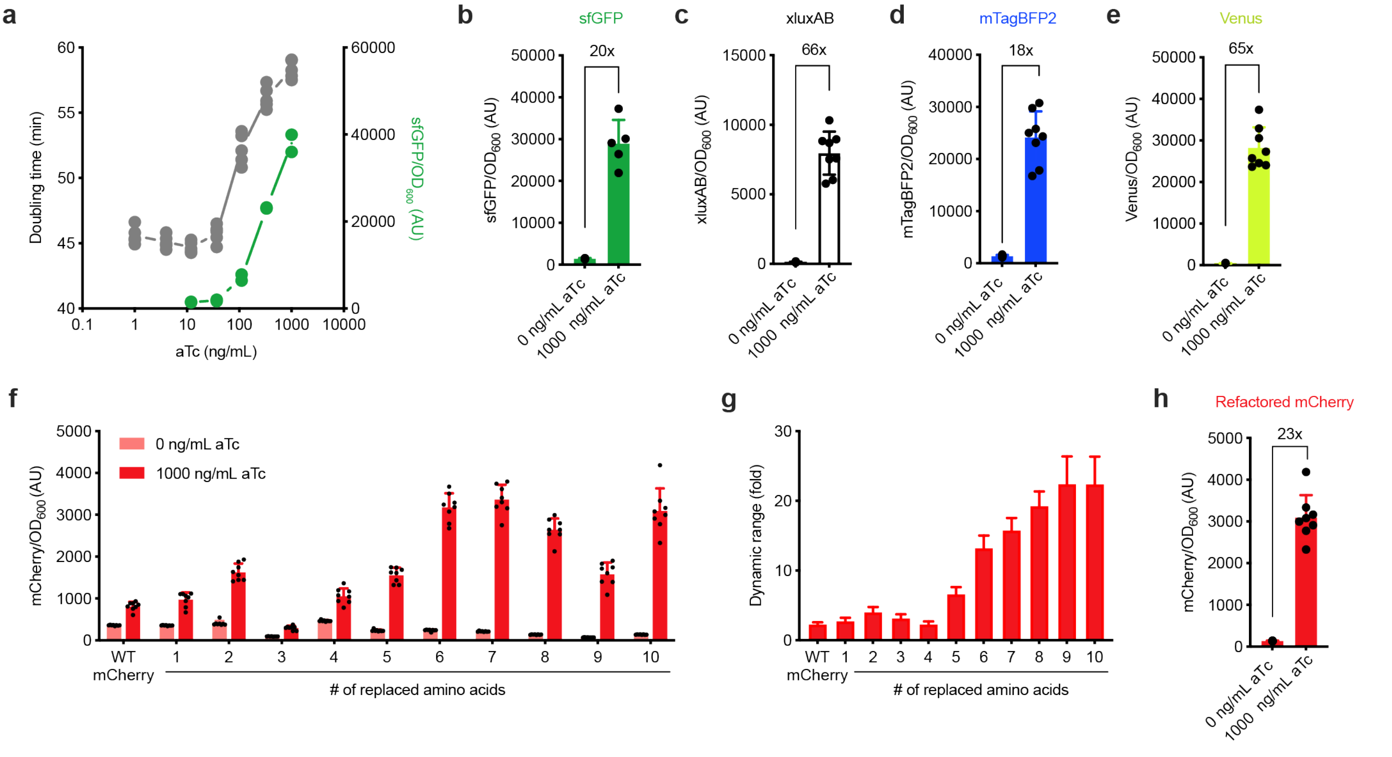

Supplementary Figure 2 | Benchmarking and extending the orthogonal translation reporter system. a) Induction of *E. coli* O-rRNA does not have a substantial effect on host growth rate. OD (optical density), n=5 and sfGFP (superfolder GFP), n=2. b) The O-sfGFP[1] reporter used throughout this study shows robust signal-to-noise upon O-rRNA induction (n=5). c-e) Additional orthogonal reporters show dynamic ranges comparable to or exceeding that of sfGFP: covalently-linked *Photorhabdus luminescens* luxAB (xluxAB)[2] (c), mTagBFP2[3] (d), and (e) Venus[4] (n=8). f-g) Conversely, an orthogonal reporter incorporating mCherry[5] showed low signal-to-noise. Replacement of successive codons at the mCherry N-terminus with their sfGFP counterparts yielded a gradual improvement in signal (f) and dynamic range (g) (n=8). h) A refactored mCherry orthogonal reporter in which the first 10 codons are replaced with the cognate sfGFP signal has improved dynamic range (n=8). Data reflect the mean and standard deviation of 2-8 biological replicates. AU = arbitrary units; aTc = anhydrotetracycline. Source data are available in the Source Data File.

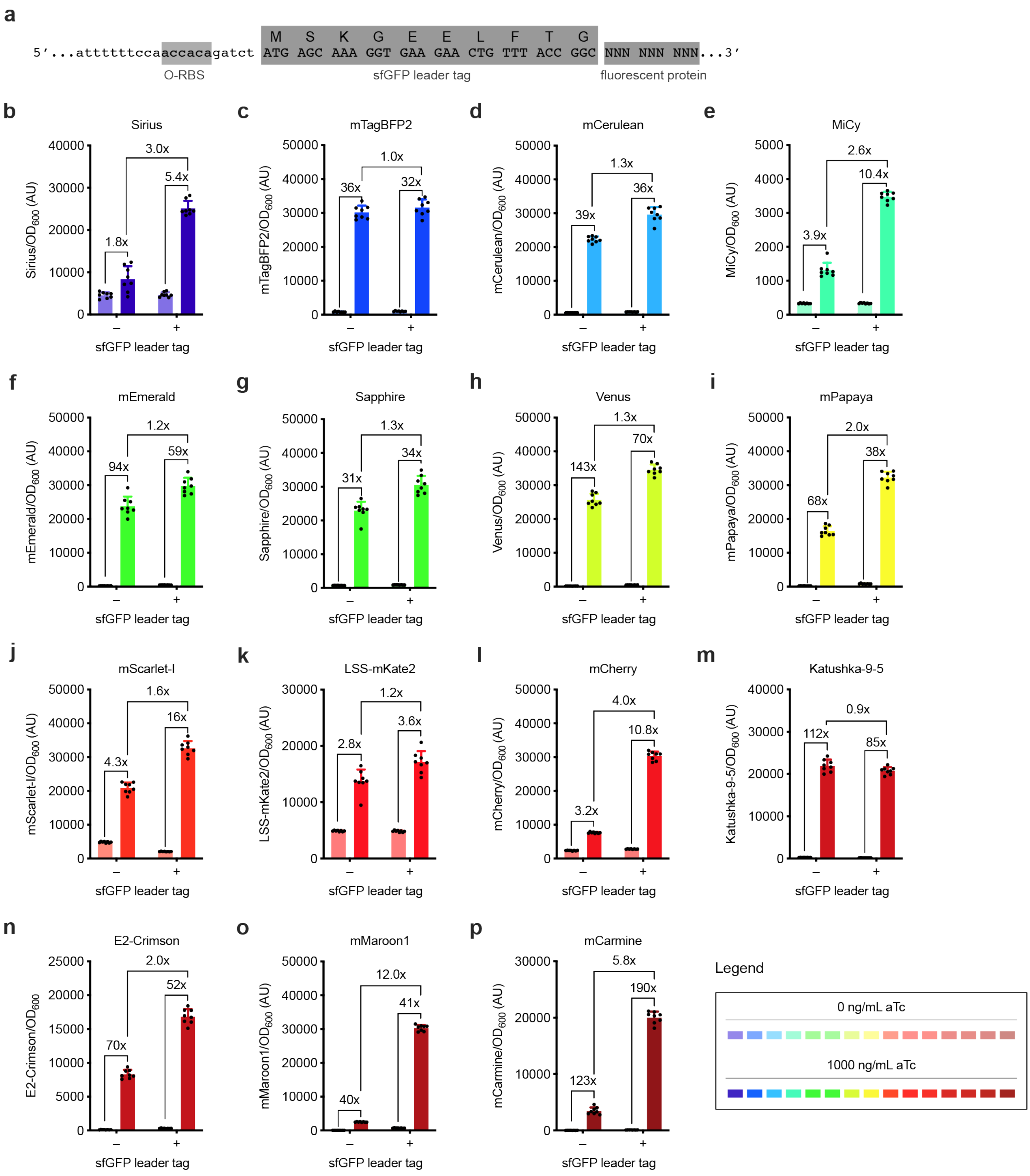

Supplementary Figure 3 | A superfolder GFP-derived leader sequence improves the function of orthogonal reporters. a) Schematic illustrating the O-RBS (orthogonal ribosome binding site), 10 amino acid sfGFP (superfolder GFP)-derived tag, and N-terminus of a fluorescent protein. When appended to the N-terminus of 15 fluorescent proteins, the tag limited reporter-dependent effects on orthogonal translation activity: b) Sirius[6], c) mTagBFP2[3], d) mCerulean[7], e) MiCy[8], f) mEmerald[9], g) Sapphire[9], h) Venus[4], i) mPapaya[10], j) mScarlet-I[11], k) LSS-mKate2[12], l) mCherry[5], m) Katusha-9-5[13], n) E2-Crimson[14], o) mMaroon1[15], p) mCarmine[16]. Generally, addition of the leader tag led to an improvement in absolute signal (average improvement 2.7-fold) and/or dynamic range (average improvement 1.5-fold). Data reflect the mean and standard deviation of 8 biological replicates. OD = optical density; AU = arbitrary units; aTc = anhydrotetracycline. Source data are available in the Source Data File.

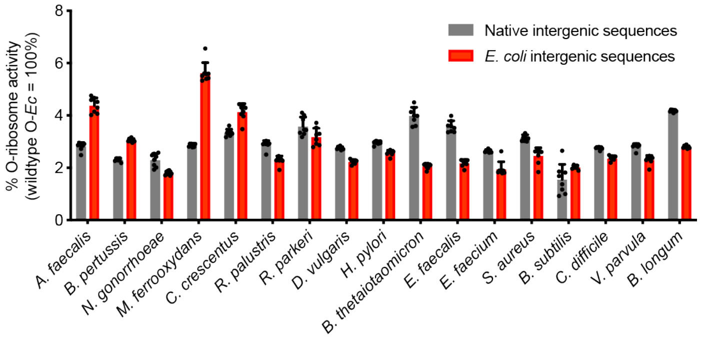

Supplementary Figure 4 | Divergent O-rRNA activities are not improved following intergenic sequence replacement. Comparison of O-sfGFP (orthogonal superfolder GFP) translation activity before and after intergenic sequence replacement for O-rRNAs derived from increasingly divergent microorganisms (69.8-82.3% 16S rRNA sequence identity to *E. coli*), showing limited improvement following intergenic sequence replacement. Data reflect the mean and standard deviation of 8 biological replicates. Comprehensive O-translation data reported in Supplementary Table 1. O-ribosome = orthogonal ribosome; wildtype O-*Ec* = wildtype orthogonal *E. coli* rRNA. Source data are available in the Source Data File.

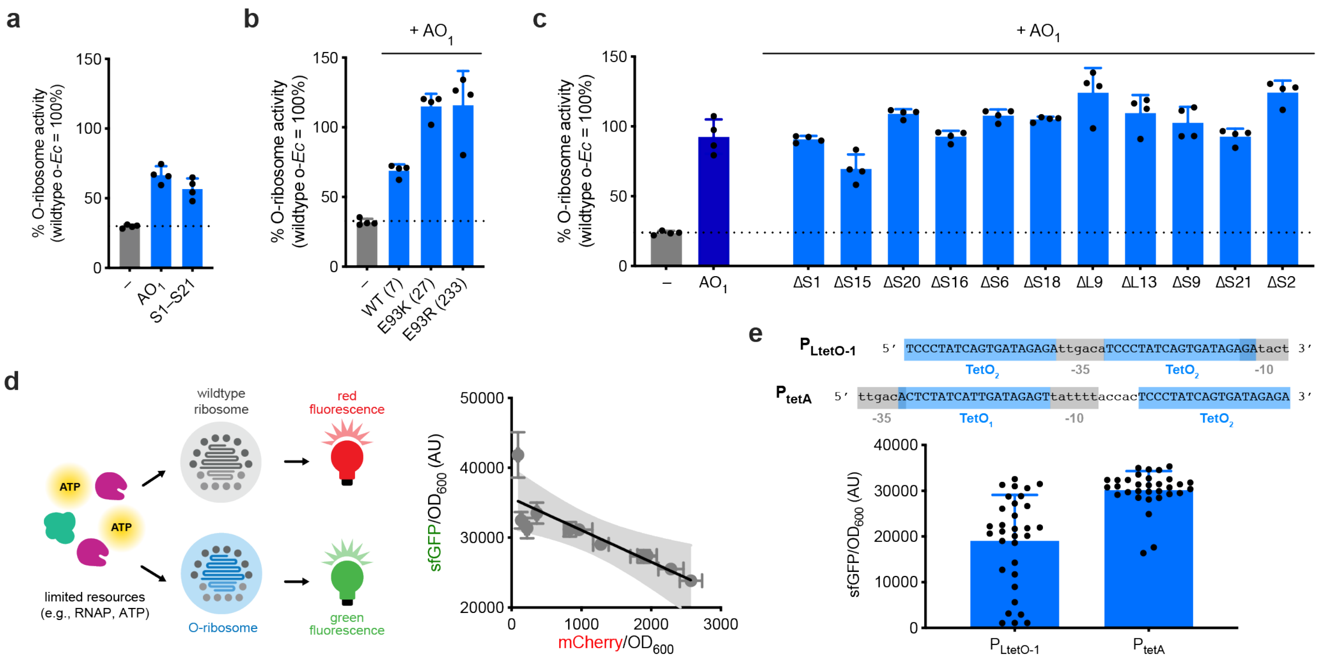

Supplementary Figure 5 | R-protein supplementation improves *A. baumannii* O-rRNA function. a) *A. baumannii* heterologous O-rRNA activity is improved following AO_1 induction, yielding comparable activity levels as supplementation with all cognate SSU (small subunit) r-proteins (S1-S21; n=4). b) *A. baumannii* heterologous O-rRNA activity improvement further depends upon AO_1 copy number, suggesting insufficient r-protein production at low copy numbers. Labels indicate RepA genotypes and numbers in parentheses indicate the approximate corresponding copy numbers[17] (n=4). c) Single r-protein deletion from AO_1 does not adversely affect *A. baumannii* heterologous O-rRNA activity, indicating that more than a single r-protein on this plasmid can complement O-rRNA function (n=4). d) O-sfGFP (orthogonal superfolder GFP) production using an *E. coli* O-rRNA is inversely proportional to mCherry production using *E. coli* native ribosomes, suggesting that r-protein overexpression may have pleiotropic effects on O-ribosome activity (99% CI, R^2 = 0.73, n=8). e) *E. coli* O-rRNA regulation using two related aTc-inducible promoters, $P_{LtetO\text{-}1}$[18] and P_{tetA}, highlights the improved signal and reduced variability using P_{tetA}, the native promoter found in the Tn10 transposon. $P_{LtetO\text{-}1}$-dependent variability is a consequence of promoter recombination between identical TetR operators (not shown) during cell passaging (n=32). Data reflect the mean and standard deviation of 4-32 biological replicates. OD = optical density; AU = arbitrary units; O-ribosome = orthogonal ribosome; wildtype O-*Ec* = wildtype orthogonal *E. coli* rRNA; ATP = adenosine triphosphate; RNAP = RNA polymerase. Source data are available in the Source Data File.

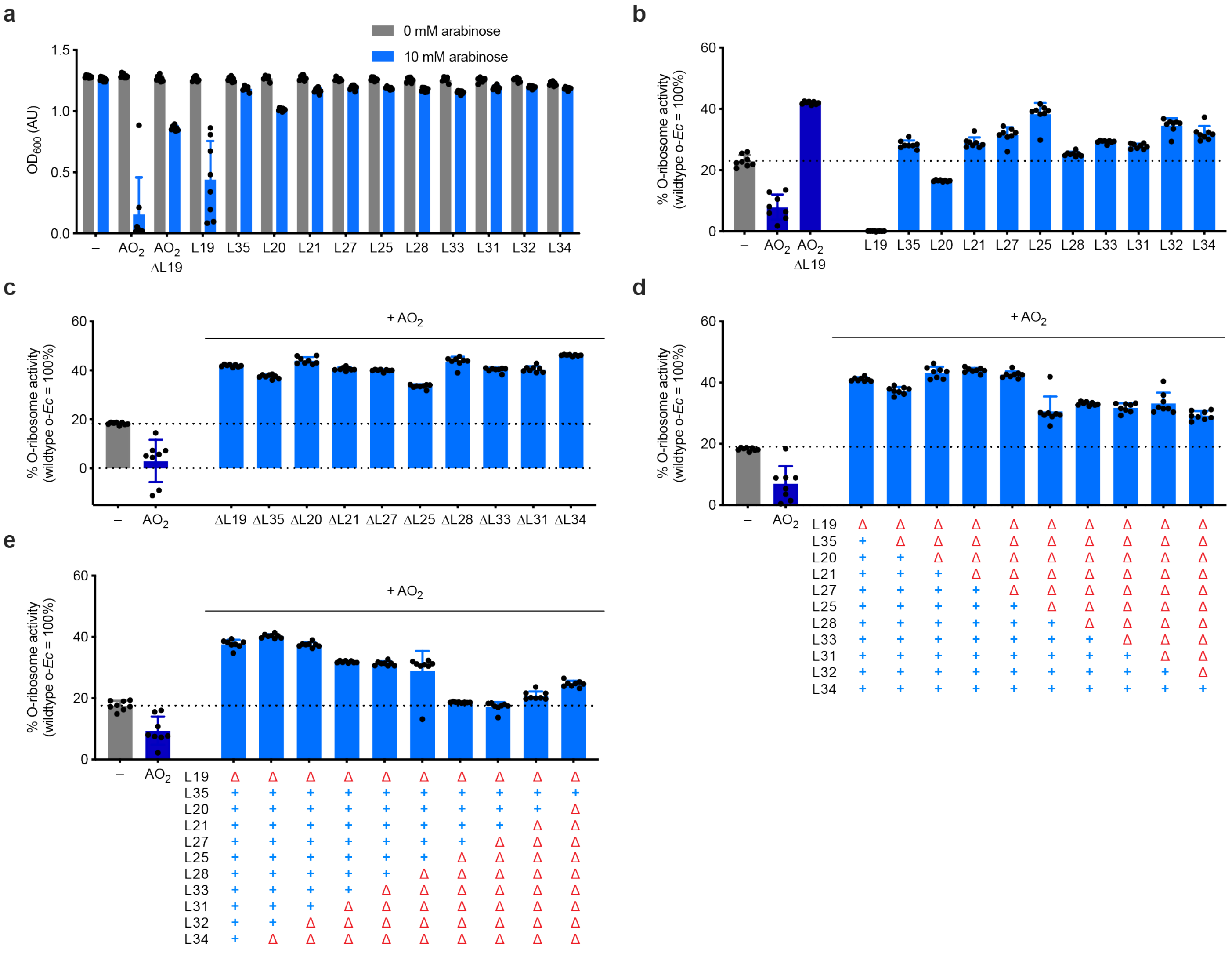

Supplementary Figure 6 | Dissection of LSU r-proteins that improve *A. macleodii* O-rRNA activity. a) Single r-proteins (from AO_2) expressed alongside cognate *A. macleodii* O-rRNA reveal that L19 is responsible for the observed toxicity from AO_2, where removal of L19 (AO_2 ΔL19) mitigates the observed growth reduction (n=7 for L25 +10 mM arabinose; otherwise n=8). b) No single r-protein from AO_2 enhances *A. macleodii* O-rRNA activity. c-e) Single deletions from AO_2 do not reveal any variants that differ in effect on O-rRNA activity, nor do truncation variants from the 5' end (d) or 3' end (e) of the artificial operon. These data collectively suggest that the observed improvement relies on the concerted action of numerous r-proteins from AO_2. Data reflect the mean and standard deviation of 8 biological replicates. OD = optical density; AU = arbitrary units; O-ribosome = orthogonal ribosome; wildtype O-*Ec* = wildtype orthogonal *E. coli* rRNA. Source data are available in the Source Data File.

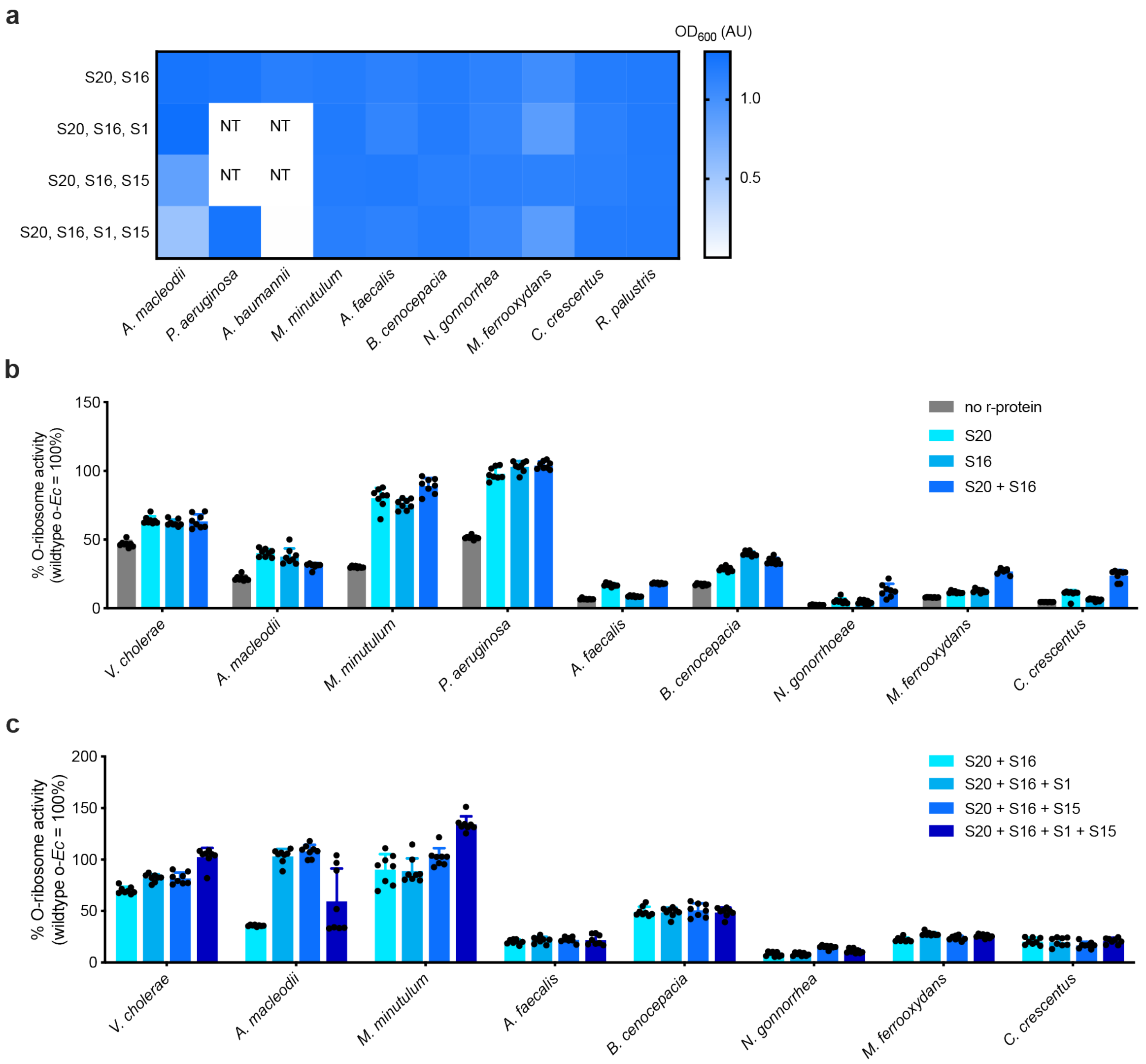

Supplementary Figure 7 | Investigation of the contributions of the identified r-proteins S20, S16, S1, and S15. a) Expression of cognate r-proteins S20, S16, S1, and S15 combinations alongside numerous heterologous O-rRNAs. *A. macleodii* and *A. baumannii* cognate S20, S16, S1, and S15 limit the growth of the *E. coli* host when co-expressed, as indicated by culture density after overnight growth, whereas most other r-proteins evaluated are well tolerated. NT = not tested. b) Both cognate r-proteins S20 and S16 are necessary for maximal sfGFP expression using O-rRNAs from more divergent microorganisms: *N. gonorrheae* (81.8% 16S rRNA sequence identity to *E. coli*), *M. ferrooxydans* (80.1%), and *C. crescentus* (79.3%). However, S20 and S16 are functionally redundant when expressed alongside more related O-rRNAs to *E. coli*. c) The combination of S20, S16, S1, and S15 is necessary for maximal activity using *V. cholerae* (90.3% 16S identity to *E. coli)* and *M. minutulum* (85.3%) O-rRNAs. For more phylogenetically distant O-rRNAs, no additional improvement is observed upon supplementation with S1 or S15 beyond the effect of S20 and S16. Data reflect the mean and standard deviation of 8 biological replicates. Comprehensive O-translation data reported in Supplementary Table 1. OD = optical density; AU = arbitrary units; O-ribosome = orthogonal ribosome; wildtype O-*Ec* = wildtype orthogonal *E. coli* rRNA. Source data are available in the Source Data File.

```
                                     h6
E. faecalis  GTCGAACGCTTCTTTCCTCCCGAGTGCTTGCACTCAATTGGAAAGAGGAGTGGCGGACGG
E. coli      GTCGAACGGTAACAGGAAGAAGCTTGCTTCTT--------TGCTGACGAGTGGCGGACGG
             ******** *           *  *****               ** *************

                                     h9/10
E. faecalis  ATACCGCATAACAGTTTATGCCGCATGGCATAAGAGTGAAAGGCGCTTTCGGGTGTCGCT
E. coli      ATACCGCATAACGTCGCAAGACCAAA---------GAGGGGGACCTTCGGGCCTCTTGCC
             ************     * * *  *          * *   * *  *   *  * * **

                                            h17
E. faecalis  CTCTGTTGTTAGAGAAGAACAAGGACGTTAGTAACTGAACGTCCCCTGACGGTATCTAAC
E. coli      TACTTTCAGCGGGGAGGAAGGGAGTAAAGTTAATACCTTTGCTCATTGACGTTACCCGCA
               ** *     * ** ***    *        *       *  *  ***** ** *

                            h26
E. faecalis  GTGCTAAGTGTTGGAGGGTTTCCGCCCTTCAGTGCTGCAGCAAACGCATTAAGCACTCCG
E. coli      CGACTTGGAGGTTGTGCCCTTGAGGC-GTGGCTTCCGGAGCTAACGCGTTAAGTCGACCG
                **  * * * * *   **  * *  *   * * * *** ***** *****    ***

                                        h44
E. faecalis  TACACACCGCCCGTCACACCACGAGAGTTTGTAACACCCGAAGTCGGTGAGGTAACCTTT
E. coli      TACACACCGCCCGTCACACCATGGGAGTGGGTTGCAAAAGAAGTAGGTAGCTTAACCTTC
             ********************* * ****  **  **   ***** ***    *******

E. faecalis  TTGGAGCCAGCCGCCTAAGGTGGGATAGATGATTGGGGTGAAGTCGTAACAAGGTAGCCG
E. coli      GG-GAGGGCGCTTACCACTTTGTGATTCATGACTGGGGTGAAGTCGTAACAAGGTAACCG
                ***   **   * *   ** ***  **** *********************** ***
```

Supplementary Figure 8 | *Enterococcus faecalis* 16S rRNA helices with low sequence identity to *E. coli*. *E. faecalis* and *E. coli* rRNAs were aligned using Clustal Omega with default parameters[19], and regions with low sequence identity were manually identified. Elements that were later transplanted into the *E. coli* 16S O-rRNA are identified in blue. h = 16S rRNA helix.

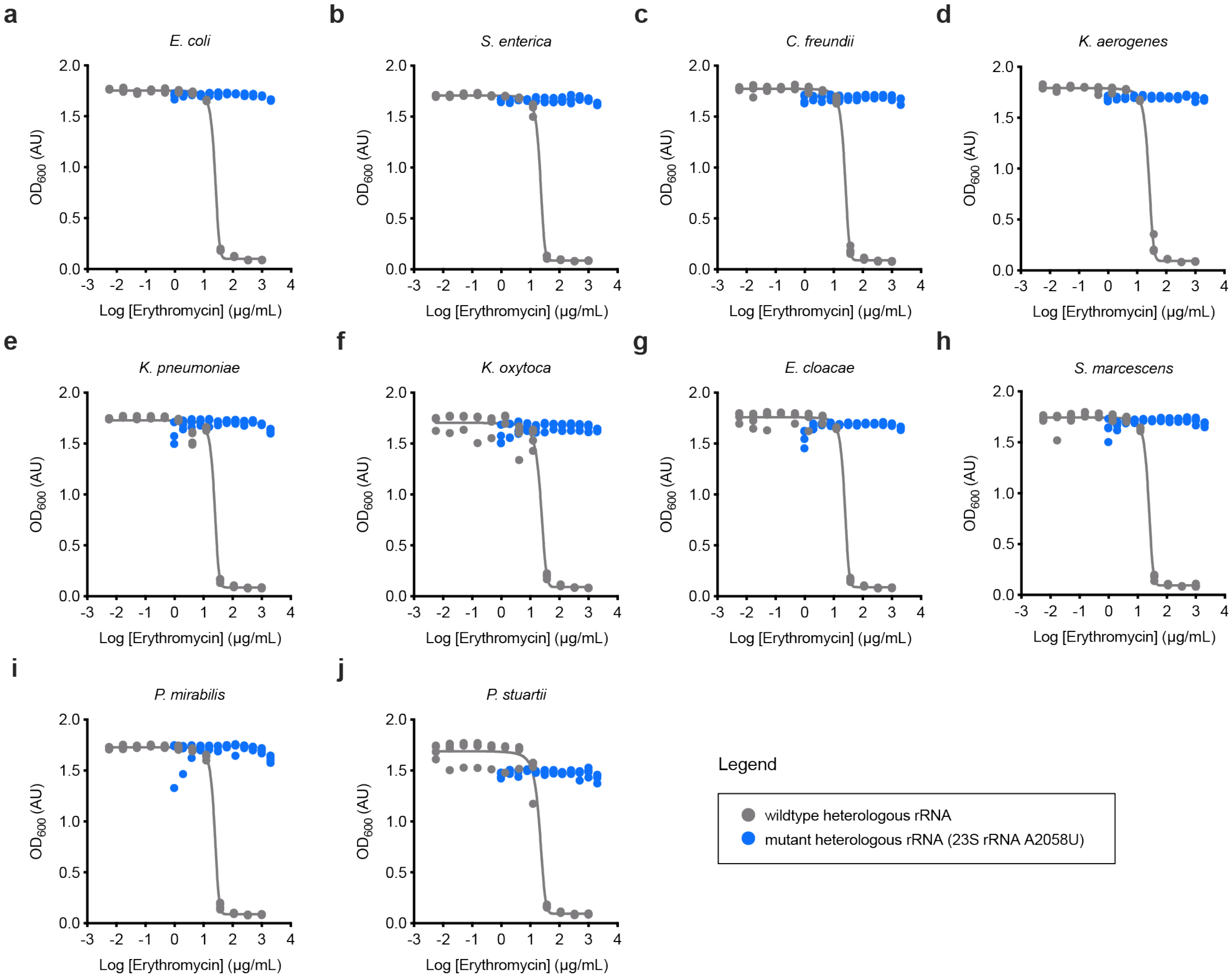

Supplementary Figure 9 | Erythromycin sensitivity of wildtype and A2058U 23S rRNAs in SQ171 cells. a-j) Erythromycin titration for SQ171 strains exclusively supported by the heterologous rRNA of interest. All strains show similar erthyromycin sensitivities using wildtype rRNAs (gray, n=4 biological replicates; best fit curves generated using GraphPad Prism version 8) or using 23S rRNA A2058U mutants (blue, n=4 biological replicates). All IC_{50} values are reported in Supplementary Table 2. OD = optical density; AU = arbitrary units. Source data are available in the Source Data File.

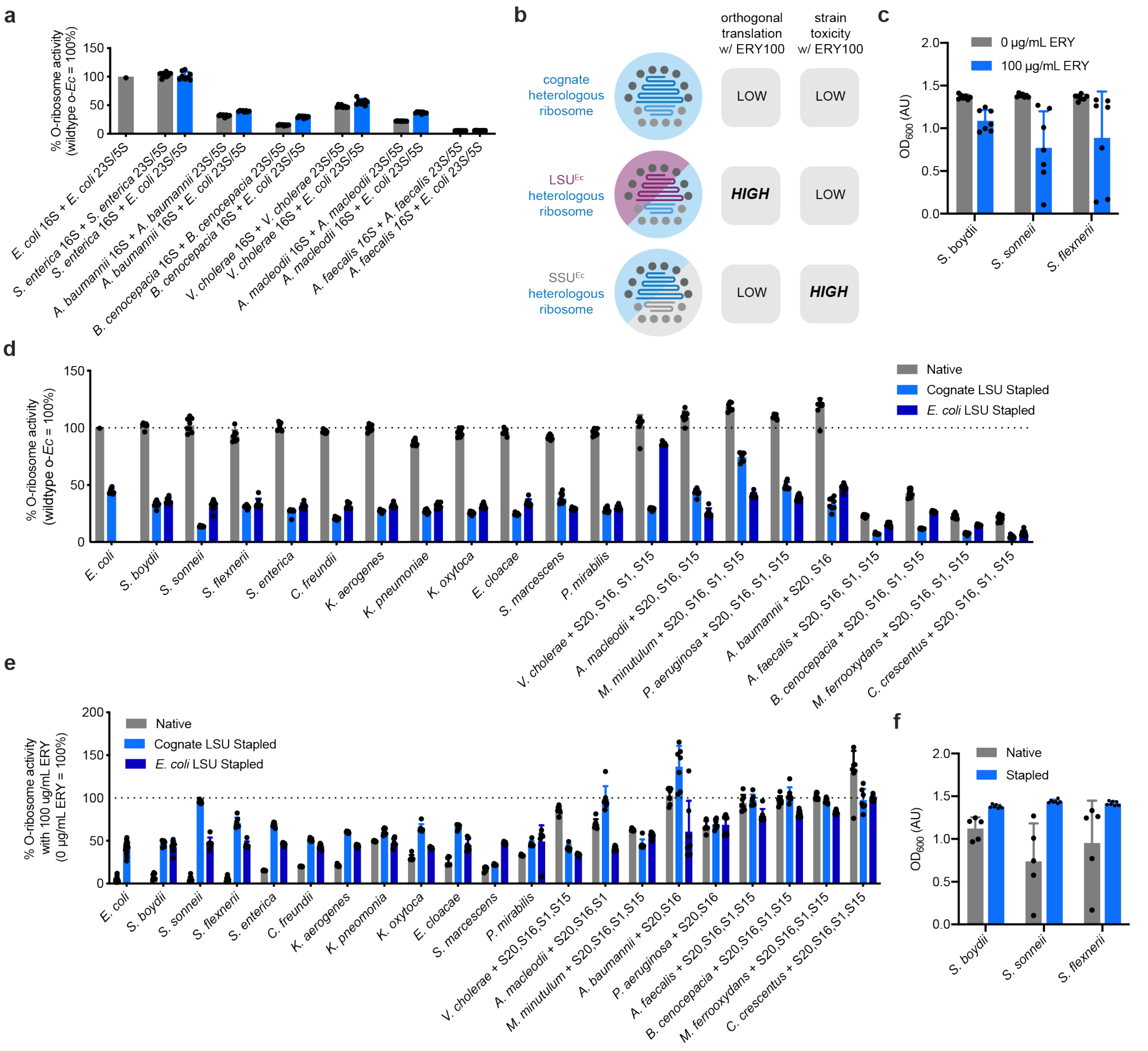

Supplementary Figure 10 | Benchmarking the ERY-dependent orthogonal translation reporter system. a) Substitution of the heterologous 23S/5S rRNAs with the *E. coli* counterparts yielded functional orthogonal translation, suggesting that exchange may occur in cases where the cognate LSU is not produced (n=8). b) The ERY (erythromycin)-dependent reporter allows discriminates between three possible subunit assembly scenarios. When an orthogonal SSU (small subunit) assembles with a cognate LSU (large subunit), the ribosome is unable to translate the orthogonal sfGFP (superfolder GFP) reporter due to ERY-sensitivity. Alternatively, heterologous SSUs may assemble with *E. coli* LSUs, resulting in robust sfGFP translation in the presence of ERY. Finally, *E. coli* SSUs may assemble with heterologous LSUs, resulting in strain toxicity due to an inability to translate essential *E. coli* genes in the presence of ERY and low sfGFP signal as a result. c) Heterologous ribosomes closely related to *E. coli* (>99.2% 16S sequence ID) re-sensitize S4246 cells to ERY treatment due to usage of sensitive LSUs for translating host genes (n=7). d) Orthogonal translation activities for native ribosomes and ribosomes stapled to cognate LSUs vs. *E. coli* LSUs (n=7 for *V. cholerae*, *E. coli* LSU stapled and *A. macleodii, E. coli* LSU stapled; otherwise n=8). e) ERY-dependent reporter data for native ribosomes and ribosomes stapled to cognate LSUs vs. *E. coli* LSUs at 100 µg mL^{-1} ERY. Data for each ribosome is normalized to its sfGFP fluorescence at 0 µg mL^{-1} ERY (n=35 for native *E. coli*, n=21 for stapled *E. coli*; otherwise n=7). f) OD_{600} for heterologous ribosomes with high 16S sequence identity to *E. coli* (≥99.2%) at 100 µg mL^{-1} ERY increases after subunit

stapling, indicating a decrease in intersubunit exchange (n=5 for native constructs, n=7 for stapled constructs). Data reflect means and standard deviations of the indicated biological replicates. Comprehensive data reported in Supplementary Table 3. OD = optical density; AU = arbitrary units; wildtype O-*Ec* = wildtype orthogonal *E. coli* rRNA. Source data are available in the Source Data File.

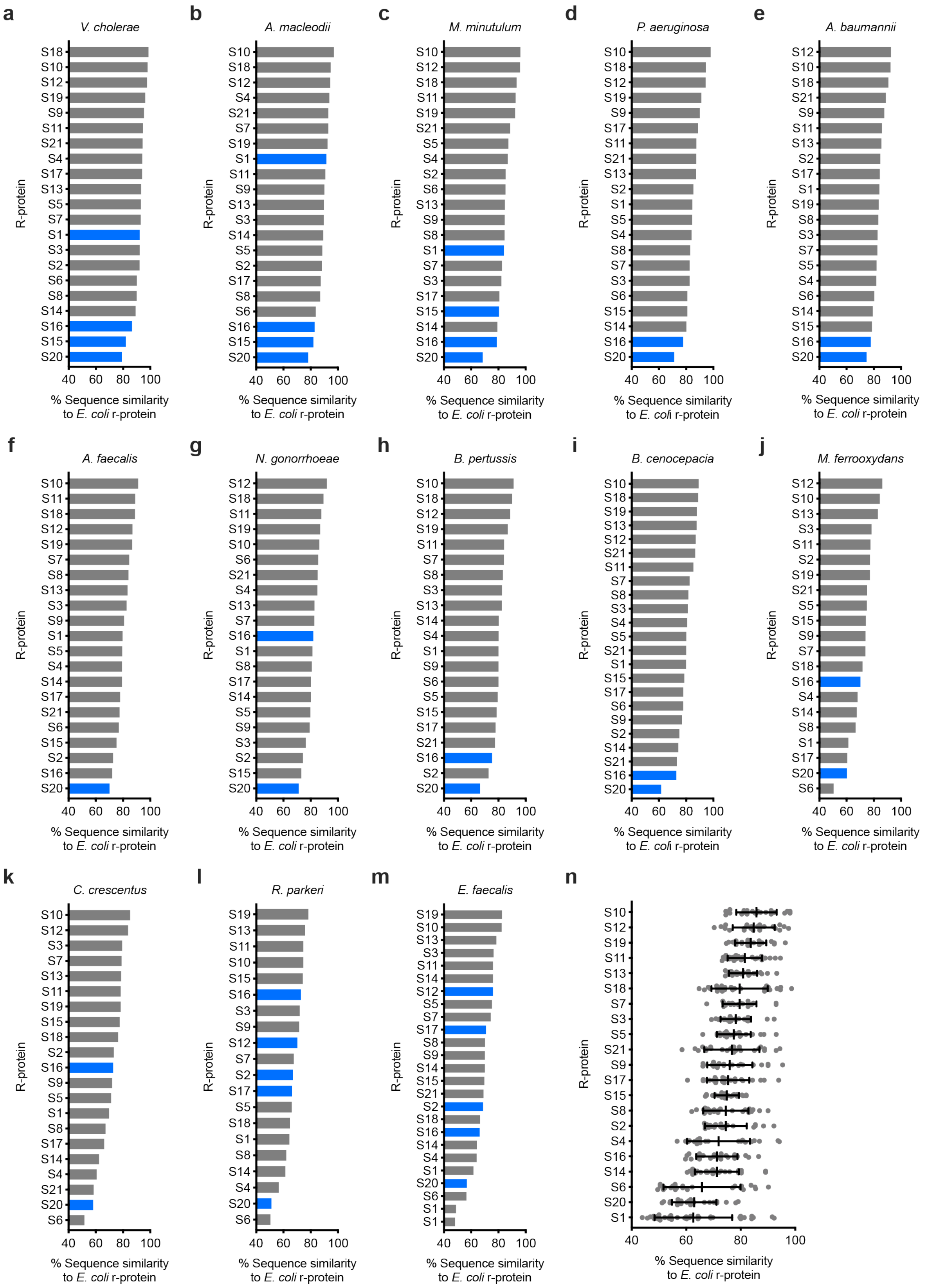

Supplementary Figure 11 | Analysis of heterologous r-protein sequence similarity to *E. coli* homologs. a-m) r-protein sequence similarities to *E. coli* for species evaluated in this study. R-proteins identified as enhancing O-rRNA activity are highlighted in blue. n) Average sequence similarity to *E. coli* for species evaluated in this study which were not immediately functional in *E. coli* prior to r-protein complementation (error bars reflect standard deviations). As protein sequences were identified via BLAST

to *E. coli* sequences (see Methods), we note that in some cases multiple homologs were identified or a full complement of r-proteins was not identified (such that n=22 for S7; n=24 for S8, S15, S17, S9, S5, S11, S19; n=25 for S18; n=29 for S16; n=37 for S1; otherwise n=23.). Source data are available in the Source Data File.

Organism Name	Bacterial Class	%16S ID	Doubling time (min)		Orthogonal translational activity (%)				
			nIS	*Ec*IS	nIS	*Ec*IS	*Ec*IS + S20/S16	*Ec*IS + S20/S16 + S1/S15	*Ec*IS + S20/S16 + S2/S12/S17
Escherichia coli	Gammaproteobacteria	100	48.1 ± 1.5 (7)	NA	100	NA	NA	NA	NA
Shigella boydii	Gammaproteobacteria	99.16	54.9 ± 2.2 (8)	43 ± 0.9 (6)	100.2 ± 3.7 (8)	102.2 ± 5.6 (8)	NT	NT	NT
Shigella sonnei	Gammaproteobacteria	99.61	46.3 ± 1.8 (8)	41.6 ± 1.2 (7)	80.6 ± 5.2 (8)	102.3 ± 7.9 (8)	NT	NT	NT
Shigella flexneri	Gammaproteobacteria	99.61	42.5 ± 2 (4)	40.9 ± 0.5 (6)	89.3 ± 4.7 (8)	93.1 ± 6.8 (8)	NT	NT	NT
Salmonella enterica	Gammaproteobacteria	97.02	43 ± 0.8 (8)	42.6 ± 1.3 (8)	102.0 ± 8.0 (8)	100.4 ± 5.8 (8)	NT	NT	NT
Citrobacter freundii	Gammaproteobacteria	96.23	47.6 ± 2 (8)	40.9 ± 1.2 (8)	61.7 ± 3.7 (8)	94.3 ± 3.6 (8)	NT	NT	NT
Klebsiella aerogenes	Gammaproteobacteria	95.84	40.1 ± 0.5 (5)	41.9 ± 2.1 (8)	94.3 ± 4.3 (8)	99.8 ± 4.3 (8)	NT	NT	NT
Klebsiella pneumoniae	Gammaproteobacteria	96.17	41.3 ± 1.1 (8)	46 ± 3.3 (8)	98.3 ± 4.7 (8)	86.2 ± 4.5 (8)	NT	NT	NT
Klebsiella oxytoca	Gammaproteobacteria	96.23	44.3 ± 3.2 (8)	43.4 ± 1.4 (8)	97.3 ± 5.1 (8)	95.8 ± 4.1 (8)	NT	NT	NT
Enterobacter cloacae	Gammaproteobacteria	97.54	44.5 ± 0.2 (3)	42.2 ± 1.1 (8)	69.8 ± 2.4 (8)	96.2 ± 2.9 (8)	NT	NT	NT
Serratia marcescens	Gammaproteobacteria	96.04	44.6 ± 1.1 (7)	42.9 ± 2.8 (6)	2.9 ± 0.2 (8)	91.8 ± 2.3 (8)	NT	NT	NT
Proteus mirabilis	Gammaproteobacteria	92.92	46.3 ± 2.3 (5)	44.5 ± 1 (6)	54.4 ± 3.8 (8)	96.1 ± 4.7 (8)	NT	NT	NT
Providencia stuartii	Gammaproteobacteria	93.12	52.5 ± 5.6 (7)	45.7 ± 1.1 (6)	14.5 ± 0.6 (8)	76.9 ± 9.4 (8)	NT	NT	NT
Vibrio cholerae	Gammaproteobacteria	90.26	56.3 ± 0.8 (6)	60.4 ± 1.4 (8)	3.3 ± 0.2 (8)	44.5 ± 2.2 (8)	60.7 ± 5.6 (8)	84.3 ± 5.7 (8)	NT
Alteromonas macleodii	Gammaproteobacteria	85.91	54.8 ± 0.9 (6)	60.2 ± 1 (4)	32.0 ± 3.1 (8)	27.8 ± 2.2 (8)	34.4 ± 2.8 (8)	61.1 ± 29.0* (8)	NT
Marinospirillum minutulum	Gammaproteobacteria	85.27	NT	NT	NT	29.5 ± 1.6 (8)	82.5 ± 5.3 (8)	118.7 ± 6.4 (8)	NT
Pseudomonas aeruginosa	Gammaproteobacteria	85.15	55.1 ± 1.8 (6)	49.9 ± 1.3 (5)	3.3 ± 0.1 (8)	57.4 ± 2.6 (8)	113.4 ± 8.0 (8)	109.9 ± 6.9 (8)	NT
Acinetobacter baumannii	Gammaproteobacteria	84.31	58.5 (2)	62.1 ± 0.8 (5)	3.4 ± 0.2 (8)	30.0 ± 4.6 (8)	117.4 ± 9.4 (8)	NG* (8)	NT
Alcaligenes faecalis	Betaproteobacteria	82.32	55 (2)	63.9 ± 10.7 (4)	2.8 ± 0.2 (8)	4.4 ± 0.4 (8)	23.1 ± 2.7 (8)	23.0 ± 1.5 (8)	NT
Bordetella pertussis	Betaproteobacteria	81.62	59 (1)	56.2 ± 4.5 (3)	2.3 ± 0.1 (8)	3.1 ± 0.2 (8)	6.0 ± 0.4 (8)	6.2 ± 0.5 (8)	NT
Burkholderia cenocepacia	Betaproteobacteria	81.45	54.5 ± 5.1 (4)	86.5 ± 2.0 (6)	1.9 ± 0.1 (8)	14.7 ± 0.6 (8)	36.7 ± 4.7 (8)	43.0 ± 4.1 (8)	28.2 ± 1.8 (8)
Neisseria gonorrhoeae	Betaproteobacteria	81.81	87.7 (1)	87.8 ± 2.7 (5)	2.3 ± 0.3 (8)	1.8 ± 0.1 (8)	8.5 ± 2.8 (8)	12 ± 1.8 (8)	NT
Mariprofundus ferrooxydans	Zetaproteobacteria	80.72	84.5 ± 4.2 (4)	63.5 ± 4.0 (3)	2.9 ± 0.1 (8)	5.6 ± 0.4 (8)	17.0 ± 0.8 (8)	22.7 ± 2.4 (8)	11.5 ± 0.5 (8)
Caulobacter crescentus	Alphaproteobacteria	79.31	NT	NT	3.3 ± 0.3 (8)	4.1 ± 0.4 (8)	20.6 ± 3.7 (8)	21.0 ± 3.1 (8)	NT
Rhodopseudomonas palustris	Alphaproteobacteria	77.55	NT	NT	2.9 ± 0.2 (8)	2.3 ± 0.2 (8)	3.3 ± 0.4 (8)	3.2 ± 0.5 (8)	NT
Rickettsia parkeri	Alphaproteobacteria	76.83	NT	NT	3.6 ± 0.4 (8)	3.2 ± 0.4 (8)	4.2 ± 0.6 (8)	4.3 ± 0.3 (8)	10.2 ± 2.2 (8)
Desulfovibrio vulgaris	Deltaproteobacteria	77.73	NT	NT	2.8 ± 0.1 (8)	2.2 ± 0.1 (8)	NT	NT	NT
Helicobacter pylori	Epsilonproteobacteria	75.13	NT	NT	3.0 ± 0.1 (8)	2.6 ± 0.1 (8)	NT	NT	NT
Bacteroides thetaiotaomicron	Bacteroidia	69.83	NT	NT	4.0 ± 0.4 (8)	2.1 ± 0.1 (8)	NT	NT	NT
Enterococcus faecalis	Bacilli	76.06	NT	NT	3.6 ± 0.3 (8)	2.2 ± 0.2 (8)	2.1 ± 0.2 (8)	NT	9.5 ± 0.6 (8)
Enterococcus faecium	Bacilli	75.98	NT	NT	2.6 ± 0.1 (8)	1.9 ± 0.3 (8)	NT	NT	NT
Staphylococcus aureus	Bacilli	76.01	NT	NT	3.1 ± 0.2 (8)	2.5 ± 0.3 (8)	NT	NT	NT
Bacillus subtilis	Bacilli	76.06	NT	NT	1.6 ± 0.6 (8)	2.0 ± 0.1 (8)	NT	NT	NT
Clostridium difficile	Clostridia	75.45	NT	NT	2.8 ± 0.1 (8)	2.4 ± 0.1 (8)	NT	NT	NT
Veillonella parvula	Clostridia	74.59	NT	NT	2.8 ± 0.2 (8)	2.3 ± 0.2 (8)	NT	NT	NT
Bifidobacterium longum	Actinobacteria	74.64	NT	NT	4.2 ± 0.2 (8)	2.8 ± 0.1 (8)	NT	NT	NT

Data reflect means and standard deviations of biological replicates indicated in parentheses (few replicates were sometimes obtained by SQ complementation; SDs are not reported for n<3).
Note that SQ data in the main text is generally reported as fitness (doublings/h), which is obtained from doubling time as described in the methods.
"NT" = not tested; NA = not applicable; NG = no growth. * indicates toxicity was observed using this combination of rRNA/r-proteins

Supplementary Table 1 | Summary of heterologous and orthogonal ribosome translation data. Doubling times in SQ171 cells (minutes) and orthogonal translation activity (normalized to orthogonal *E. coli*) for all heterologous ribosomes tested. nIS = native intergenic sequences; *Ec*IS = *E. coli* intergenic sequences. NA = not applicable; NT = not tested.

Organism Name	ERY IC_{50} (µg/mL)	
	Wildtype 23S rRNA	A2058U 23S rRNA
Escherichia coli	20.8 ± 1.02	>2000
Salmonella enterica	18.82 ± 1.02	>2000
Citrobacter freundii	20.83 ± 1.02	>2000
Klebsiella aerogenes	21.4 ± 1.02	>2000
Klebsiella pneumoniae	20.17 ± 1.05	>2000
Klebsiella oxytoca	19.66 ± 1.06	>2000
Enterobacter cloacae	19.75 ± 1.06	>2000
Serratia marcescens	19.98 ± 1.03	>2000
Proteus mirabilis	20.57 ± 1.02	>2000
Providencia stuartii	18.35 ± 1.06	>2000

Supplementary Table 2 | Tabulated erythromycin IC_{50} values for wildtype and 23S rRNA A2058U rRNAs. Data reflect means and standard deviations of 4 biological replicates. Data are plotted in Supplementary Figure 9. rRNA = ribosomal RNA; ERY = erythromycin; IC_{50} = half maximal inhibitory concentration.

Organism Name	Bacterial Class	%16S rRNA identity	Orthogonal Translational Activity (%)			% O-ribosome Activity at 100 μg/mL ERY (0 μg/mL ERY = 100%)		
			Native	Cognate LSU Stapled	*E. coli* LSU Stapled	Native	Cognate LSU Stapled	*E. coli* LSU Stapled
Escherichia coli	Gammaproteobacteria	100	100	44.4 ± 2.7	NA	3.5 ± 2.7	41.3 ± 7.3	NA
Shigella boydii	Gammaproteobacteria	99.16	100.2 ± 3.7	33.0 ± 2.7	36.3 ± 2.8	8.1 ± 3.6	47.0 ± 1.8	42.9 ± 7.0
Shigella sonnei	Gammaproteobacteria	99.61	80.6 ± 5.2	13.8 ± 0.7	31.6 ± 4.5	4.8 ± 3.1	95.9 ± 1.9	47.1 ± 6.7
Shigella flexneri	Gammaproteobacteria	99.61	89.3 ± 4.7	31.0 ± 1.7	33.8 ± 4.3	4.7 ± 3.0	71.0 ± 6.0	45.2 ± 3.9
Salmonella enterica	Gammaproteobacteria	97.02	102.0 ± 8.0	6.6 ± 2.9	31.5 ± 2.7	15.3 ± 0.4	69.2 ± 2.6	46.3 ±1.8
Citrobacter freundii	Gammaproteobacteria	96.23	61.7 ± 3.7	20.8 ± 1.7	31.8 ± 2.7	20.1 ± 0.4	51.4 ± 1.6	43.5 ± 2.7
Klebsiella aerogenes	Gammaproteobacteria	95.84	94.3 ± 4.3	27.2 ± 1.8	31.7 ± 2.1	21.2 ±1.3	60.2 ± 1.1	44.1 ± 1.5
Klebsiella pneumoniae	Gammaproteobacteria	96.17	98.3 ± 4.7	26.8 ± 1.6	32.1 ± 2.8	49.4 ± 0.6	60.7 ± 3.8	47.4 ± 4.9
Klebsiella oxytoca	Gammaproteobacteria	96.23	97.3 ± 5.1	25.5 ± 1.6	31.1 ± 2.4	29.8 ± 3.5	64.6 ± 5.0	41.8 ± 1.1
Enterobacter cloacae	Gammaproteobacteria	97.54	69.8 ± 2.4	24.7 ± 1.6	34.4 ± 3.2	25.8 ± 5.0	66.5 ± 2.7	45.3 ± 6.7
Serratia marcescens	Gammaproteobacteria	96.04	2.9 ± 0.2	38.4 ± 2.7	29.2 ± 1.1	17.3 ± 2.6	22.0 ± 0.7	47.5 ± 1.6
Proteus mirabilis	Gammaproteobacteria	92.92	54.4 ± 3.8	28.4 ± 2.7	30.0 ± 2.0	33.0 ± 0.8	46.4 ± 3.3	56.5 ± 3.3
Vibrio cholerae + S20, S16, S1, S15	Gammaproteobacteria	90.26	84.3 ± 5.7	29.1 ± 1.5	77.8 ± 4.6	85.6 ± 4.4	41.9 ± 3.3	34.0 ± 1.3
Alteromonas macleodii + S20, S16, S15	Gammaproteobacteria	85.91	109.5 ± 5.3	36.1 ± 2.7	17.3 ± 3.8	69.5 ± 5.7	99.2 ± 14.6	41.3 ± 1.7
Marinospirillum minutulum + S20, S16, S1, S15	Gammaproteobacteria	85.27	118.7 ± 6.4	67.9 ± 4.9	39.4 ± 3.7	62.9 ± 5.7	46.0 ± 5.8	55.7 ± 3.9
Pseudomonas aeruginosa + S20, S16, S1, S15	Gammaproteobacteria	85.15	109.9 ± 6.9	47.2 ± 4.9	39.3 ± 4.3	103.1 ± 9.5	137.0 ± 23.8	60.9 ± 35.8
Acinetobacter baumannii + S20, S16	Gammaproteobacteria	84.31	117.4 ± 9.4	33.4 ± 4.9	47.4 ± 3.1	66.7 ± 6.5	69.4 ± 5.4	69.2 ± 10.8
Alcaligenes faecalis + S20, S16, S1, S15	Betaproteobacteria	82.32	23.0 ± 1.5	7.3 ± 0.7	16.0 ± 1.9	94.5 ± 9.3	97.4 ± 6.5	79.5 ± 7.8
Burkholderia cenocepacia + S20, S16, S1, S15	Betaproteobacteria	81.45	43.0 ± 4.1	11.8 ± 0.5	26.4 ± 1.0	96.5 ± 5.6	103.2 ± 9.3	81.6 ± 3.9
Mariprofundus ferrooxydans + S20, S16, S1, S15	Zetaproteobacteria	80.72	22.7 ± 2.4	7.5 ± 1.0	12.1 ± 1.0	102.0 ± 9.5	96.6 ± 2.8	82.9 ± 2.3
Caulobacter crescentus + S20, S16, S1, S15	Alphaproteobacteria	79.31	21.0 ± 3.1	3.8 ± 2.6	5.8 ± 2.0	129.6 ± 25.5	98.0 ± 12.7	99.0 ± 3.2

Supplementary Table 3 | Summary of stapled heterologous ribosome experiments. Data reflect means and standard deviations of 7-28 biological replicates. ERY = erythromycin; rRNA = ribosomal RNA; LSU = large subunit.

Name	Function	Sequence
AB3441	Universal rRNA amplification primer, FWD	ACCGGCCGCUGTGCCAGCAGCCGCGGTAATAC
AB3442	Universal rRNA amplification primer, REV	AGGGGTTCCGCGCACAUGTGACGGGCGGTGTGTACAAG
AB5708	Recombineering primer, rrlA-H A2058U	C*T*C*A*ATGTTCAGTGTCAAGCTATAGTAAAGGTTCACGGGGTCTTACCGTCTTGCCGCGGGTACACTGCATCTTCACAGCGAGTTCAATTT
AB5710	Recombineering check primer, FWD	GAAATTCCTTGTCGGGTAAGTTCC
AB5711	Recombineering check primer, REV	GAACATCAAACATTAAAGGGTGGTATTTC

blue = USER junction; red = annealing region; *=phosphothirate bonds; yellow = mutated base

Supplementary Table 4 | Primers used in this study. Instances where primers haven been used are described in the Methods section. rRNA = ribosomal RNA; FWD = forward; REV = reverse.

Reporter Protein	λ_{Ex} (nm)	λ_{Em} (nm)	sfGFP Leader Tag	
			–	+
Sirius	355	424	pNK141a1	pNK141a2
mTagBFP2	399	454	pNK133b	pNK141b2
mCerulean	433	475	pNK141c1	pNK141c2
MiCy	472	495	pNK141m1	pNK141m2
mEmerald	487	509	pNK141e1	pNK141e2
Sapphire	399	511	pNK141d1	pNK141d2
Venus	515	530	pNK133d	pNK141f2
mPapaya	530	550	pNK141g1	pNK141g2
mScarlet-I	569	593	pNK141h1	pNK141h2
LSS-mKate2	460	605	pNK141l1	pNK141l2
mCherry	587	610	pNK133a	pNK141i2
Katushka-9-5	588	635	pNK141j1	pNK141j2
E2-Crimson	611	646	pNK141n1	pNK141n2
mMaroon1	609	657	pNK141o1	pNK141o2
mCarmine	603	675	pNK141k1	pNK141k2
ermCL-sfGFP	485	510	pAB140j8	N/A
sfGFP	485	510	Plpp5.B.GFP	N/A

Supplementary Table 5 | Reporter plasmids used in this study. Highlighted plasmids (blue) have been deposited in Addgene. Addgene IDs are listed in Supplementary Table 8. Plasmid Plpp5.B.GFP was kindly provided by the Jewett Lab. sfGFP = superfolder GFP; λ_{Ex} = excitation wavelength; λ_{Em} = emission wavelength.

Organism Name	NCBI TaxID	NCBI Species TaxID	NCBI Strain Identifier	Accession (GTDB Genome Representative)
Acinetobacter baumannii	575584	470	ATCC 19606	RS_GCF_002811175.1
Alcaligenes faecalis	511	511	ZD02	RS_GCF_000967305.2
Alteromonas macleodii	529120	28108	ATCC 27126	RS_GCF_000172635.2
Burkholderia cenocepacia	331272	95486	HI2424	RS_GCF_000203955.1
Bifidobacterium longum	206672	216816	NCC2705	RS_GCF_000007525.1
Bordetella pertussis	257313	520	Tohama I	RS_GCF_000195715.1
Bacillus subtilis	224308	1423	168	RS_GCF_000009045.1
Bacteroides thetaiotaomicron	818	818	7330	RS_GCF_001314975.1
Caulobacter crescentus	565050	155892	NA1000	RS_GCF_000022005.1
Clostridium difficile	272563	1496	630	RS_GCF_000009205.1
Citrobacter freundii	546	546	CAV1321	RS_GCF_001022155.1
Desulfovibrio vulgaris	882	881	Hildenborough	RS_GCF_000195755.1
Enterobacter cloacae	716541	550	ATCC 13047	RS_GCF_000025565.1
Escherichia coli	511145	562	K-12	RS_GCF_000005845.2
Escherichia coli/Shigella Spp.	198214	623	301	RS_GCF_000006925.2
Enterococcus faecalis	226185	1351	V583	RS_GCF_000007785.1
Enterococcus faecium	333849	1352	DO	RS_GCF_000174395.2
Helicobacter pylori	210	210	KH33	RS_GCF_002832255.1
Klebsiella aerogenes	1028307	548	KCTC 2190	RS_GCF_000215745.1
Klebsiella oxytoca	571	571	CAV1015	RS_GCF_001870185.1
Klebsiella pneumoniae	1125630	573	HS11286	RS_GCF_000240185.1
Mariprofundus ferrooxydans	314345	314344	PV-1	RS_GCF_000153765.1
Neisseria gonorrhoeae	242231	485	FA 1090	RS_GCF_000006845.1
Pseudomonas aeruginosa	208964	287	PAO1	RS_GCF_000006765.1
Proteus mirabilis	529507	584	HI4320	RS_GCF_000069965.1
Providencia stuartii	1157951	588	MRSN 2154	RS_GCF_000259175.1
Rhodopseudomonas palustris	316058	1076	HaA2	RS_GCF_000013365.1
Rickettsia parkeri	1105108	35792	Portsmouth	RS_GCF_000284195.1
Staphylococcus aureus	93061	1280	NCTC 8325	RS_GCF_000013425.1
Salmonella enterica	90371	28901	LT2	RS_GCF_002289225.1
Serratia marcescens	911022	615	ATCC 13880	RS_GCF_000735445.1
Vibrio cholerae	243277	666	N16961	RS_GCF_000006745.1
Veillonella parvula	479436	29466	DSM 2008	RS_GCF_000024945.1

Supplementary Table 6 | Species names and GTDB representative genomes used to construct phylogenetic tree.

	rRNA Expression Plasmids						R-Protein Expression Plasmids										
							Individual R-proteins				R-protein Operons						
**Intergenic Regions**	Native	*E. coli*	Native	*E. coli*	*E. coli*	*E. coli*											
**Anti-Ribosome Binding Site**	Wildtype	Wildtype	*Anti-B*	*Anti-B*	*Anti-B*	*Anti-B*											
**Stapled vs. WT**	WT	WT	WT	WT	Stapled	Stapled	**S20, S16**	**S20, S16, S15**	**S20, S16, S1, S15**	**S2, S12, S20, S16, S17**	**alpha (α)**	**beta (β)**	**str**	**spc**	**S10**	**AO1**	**AO2**
**Co-Produced LSU**	Cognate	Cognate	Cognate	*Cognate*	Cognate	*E. coli*											
Shigella sonnei	pRF001a	pRF001b	pRF001c	pRF001d	pRF112	pRF112a	-	-	-	-	-	-	-	-	-	-	-
Shigella flexneri	pRF002a	pRF002b	pRF002c	pRF002d	pRF113	pRF113a	-	-	-	-	-	-	-	-	-	-	-
Escherichia coli	pSB19b	pSB19b	pAB172a	pAB172a	pRF100	pRF100	-	-	-	-	-	-	-	-	-	-	-
Salmonella enterica	pRF003a	pRF003b	pRF003c	pNK007p	pRF114	pRF114a	-	-	-	-	-	-	-	-	-	-	-
Citrobacter freundii	pRF004a	pRF004b	pRF004c	pRF004d	pRF115	pRF115a	-	-	-	-	-	-	-	-	-	-	-
Klebsiella aerogenes	pRF005a	pRF005b	pRF005c	pRF005d	pRF116	pRF116a	pNK143	-	-	-	-	-	-	-	-	-	-
Klebsiella pneumoniae	pRF006a	pRF006b	pRF006c	pRF006d	pRF117	pRF117a	pNK144	-	-	-	-	-	-	-	-	-	-
Klebsiella oxytoca	pRF007a	pRF007b	pRF007c	pRF007d	pRF118	pRF118a	-	-	-	-	-	-	-	-	-	-	-
Enterobacter cloacae	pRF008a	pRF008b	pRF008c	pRF008d	pRF119	pRF119a	-	-	-	-	-	-	-	-	-	-	-
Serratia marcescens	pRF009a	pRF009b	pRF009c	pNK041p	pAB207a	pAB207a2	-	-	-	-	-	-	-	-	-	-	-
Proteus mirabilis	pRF010a	pRF010b	pRF010c	pRF010d	pAB207b	pAB207b2	-	-	-	-	-	-	-	-	-	-	-
Providencia stuartii	pRF011a	pRF011b	pRF011c	pNK059p	-	-	pNK083g	-	-	-	-	-	-	-	-	-	-
Vibrio cholerae	pRF012a	pRF012b	pRF012c	pNK013p	pAB207c	pAB207c2	pNK083f	-	pAB184h	-	pNK096c	pNK096e	pNK096d	pNK096b	pNK096a	pNK096f	pNK096g
Alteromonas macleodii	pRF013a	pRF013b	pRF013c	pNK045p	pAB207d	pAB207d2	pNK083e	pNK108h	pNK102c	-	pNK099c	pNK099e	pNK099d	pNK099b	pNK099a	pNK099f	pNK099g
Marinospirillum minutulum	pNK112a	pNK112b	pNK112c	pAC027	pAB207e	pAB207e2	pAB184e	-	pAB184e2	-	-	-	-	-	-	-	-
Pseudomonas aeruginosa	pRF014a	pRF014b	pRF014c	pNK003p	pAB207f	pAB207f2	pNK083d	-	pNK136a	-	-	-	-	-	-	-	-
Acinetobacter baumannii	pRF015a	pRF015b	pRF015c	pAC018	pRF101	pRF101a	pNK083b	-	pNK136b	-	pAB164b3	pAB164b5	pAB164b4	pAB164b2	pAB164b	pAB164b6	pAB164b7
Alcaligenes faecalis	pRF016a	pRF016b	pRF016c	pAC044	pAB207g	pAB207g2	pAB184c	-	pAB184c3	-	pAB164c3	pAB164c5	pAB164c4	pAB164c2	pAB164c	pAB164c6	pAB164c7
Bordetella pertussis	pRF017a	pRF017b	pRF017c	pAC037	-	-	pAB184b	-	pAB184b3	-	-	-	-	-	-	-	-
Burkholderia cenocepacia	pRF018a	pRF018b	pRF018c	pNK053p	pRF102	pRF102a	pNK083c	-	pAB184g	-	pNK098c	pNK098e	pNK098d	pNK098b	pNK098a	pNK098f	pNK098g
Neisseria gonorrhoeae	pRF019a	pRF019b	pRF019c	pAC036	-	-	pAB184a	-	pAB184a3	-	-	-	-	-	-	-	-
Mariprofundus ferrooxydans	pRF020a	pRF020b	pRF021c	pAC030	pAB207h	pAB207h2	pAB184f	-	pAB184f2	-	-	-	-	-	-	-	-
Caulobacter crescentus	-	-	pRF022c	pAC042	pAB207i	pAB207i2	pNK131a	-	pNK131d	-	-	-	-	-	-	-	-
Rhodopseudomonas palustris	-	-	pRF023c	pAC038	-	-	pNK132a	-	pNK132d	-	-	-	-	-	-	-	-
Rickettsia parkeri	-	-	pRF024c	pAC040	-	-	pAB184d	-	pAB184d3	pNK138b	pAB185c	pAB185e	pAB185d	pAB185b	pAB185a	pAB185f	pAB185g
Desulfovibrio vulgaris	-	-	pRF025c	pAC039	-	-	-	-	-	-	-	-	-	-	-	-	-
Helicobacter pylori	-	-	pRF027c	pAC041	-	-	-	-	-	-	-	-	-	-	-	-	-
Bacteroides thetaiotaomicron	-	-	pRF028c	pRF028d	-	-	-	-	-	-	-	-	-	-	-	-	-
Enterococcus faecalis	-	-	pRF029c	pAC019	-	-	pNK155	-	-	pNK078a	-	-	-	-	-	-	-
Enterococcus faecium	-	-	pRF030c	pRF030d	-	-	-	-	-	-	-	-	-	-	-	-	-
Staphylococcus aureus	-	-	pRF031c	pRF031d	-	-	-	-	-	-	-	-	-	-	-	-	-
Bacillus subtilis	-	-	pRF032c	pRF032d	-	-	-	-	-	-	-	-	-	-	-	-	-
Clostridium difficile	-	-	pRF033c	pRF033d	-	-	-	-	-	-	-	-	-	-	-	-	-
Veillonella parvula	-	-	pRF034c	pRF034d	-	-	-	-	-	-	-	-	-	-	-	-	-
Bifidobacterium longum	-	-	pRF035c	pRF035d	-	-	-	-	-	-	-	-	-	-	-	-	-
Mycobacterium tuberculosis	-	-	pNK036c	pRF036d	-	-	-	-	-	-	-	-	-	-	-	-	-

Supplementary Table 7 | rRNA and r-protein expression plasmids used in this study. rRNA/r-protein combinations yielding the highest degree of activity have been deposited in Addgene and are highlighted in blue. Addgene IDs are listed in Supplementary Table 8. rRNA = ribosomal RNA; r-protein = ribosomal protein; LSU = large subunit.

rRNA Plasmids	
Name	Addgene ID
pNK110d	156321
pRF001d	156322
pRF002d	156323
pAB172a	156324
pNK007p	156325
pRF004d	156326
pRF005d	156327
pRF006d	156328
pRF007d	156329
pRF008d	156330
pNK041p	156331
pRF010d	156332
pNK059p	156333
pNK013p	156334
pNK045p	156335
pAC027	156336
pNK003p	156337
pAC018	156338
pAC044	156339
pAC037	156340
pNK053p	156341
pAC036	156342
pAC030	156343
pAC042	156344
pAC038	156345
pAC040	156346
pAC039	156347
pAC041	156348
pRF028d	156349
pAC019	156350
pRF030d	156351
pRF031d	156352
pRF032d	156353
pRF033d	156354
pRF034d	156355
pRF035d	156356
pRF036d	156357

R-protein Plasmids	
Name	Addgene ID
pAB184a	156358
pAB184b3	156359
pAB184c3	156360
pAB184d3	156361
pAB184e2	156362
pAB184f2	156363
pAB184g	156364
pAB184h	156365
pNK083b	156366
pNK083d	156367
pNK108h	156368
pNK131d	156369
pNK132d	156370
pNK055b	156371

Reporter Plasmids	
Name	Addgene ID
pAB140j8	156373
pNK141a2	156374
pNK141b2	156375
pNK141c2	156376
pNK141d2	156377
pNK141e2	156378
pNK141f2	156379
pNK141g2	156380
pNK141h2	156381
pNK141i2	156382
pNK141j2	156383
pNK141k2	156384
pNK141l2	156385
pNK141m2	156386
pNK141n2	156387
pNK141o2	156388

Strains	
Name	Addgene ID
S4246	156372

Supplementary Table 8 | Addgene IDs of deposited plasmids.

SUPPLEMENTARY REFERENCES

1. Pedelacq JD, Cabantous S, Tran T, Terwilliger TC, Waldo GS. Engineering and characterization of a superfolder green fluorescent protein. *Nat Biotechnol* **24**, 79-88 (2006).

2. Carlson JC, Badran AH, Guggiana-Nilo DA, Liu DR. Negative selection and stringency modulation in phage-assisted constinuous evolution. *Nat Chem Biol* **10**, 216-222 (2014).

3. Subach OM, Cranfill PJ, Davidson MW, Verkhusha VV. An enhanced monomeric blue fluorescent protein with the high chemical stability of the chromophore. *PLoS One* **6**, e28674 (2011).

4. Nagai T, Ibata K, Park ES, Kubota M, Mikoshiba K, Miyawaki A. A variant of yellow fluorescent protein with fast and efficient maturation for cell-biological applications. *Nat Biotechnol* **20**, 87-90 (2002).

5. Shaner NC, Campbell RE, Steinbach PA, Giepmans BNG, Palmer AE, Tsien RY. Improved monomeric red, orange and yellow fluorescent proteins derived from Discosoma sp. red fluorescent protein. *Nature Biotechnology* **22**, 1567-1572 (2004).

6. Tomosugi W*, et al.* An ultramarine fluorescent protein with increased photostability and pH insensitivity. *Nature Methods* **6**, 351-353 (2009).

7. Rizzo MA, Piston DW. High-contrast imaging of fluorescent protein FRET by fluorescence polarization microscopy. *Biophys J* **88**, L14-16 (2005).

8. Karasawa S, Araki T, Nagai T, Mizuno H, Miyawaki A. Cyan-emitting and orange-emitting fluorescent proteins as a donor/acceptor pair for fluorescence resonance energy transfer. *Biochem J* **381**, 307-312 (2004).

9. Cubitt AB, Woollenweber LA, Heim R. Understanding structure-function relationships in the Aequorea victoria green fluorescent protein. *Methods Cell Biol* **58**, 19-30 (1999).

10. Hoi H*, et al.* An engineered monomeric Zoanthus sp. yellow fluorescent protein. *Chem Biol* **20**, 1296-1304 (2013).

11. Bindels DS*, et al.* mScarlet: a bright monomeric red fluorescent protein for cellular imaging. *Nature Methods* **14**, 53-56 (2016).

12. Piatkevich KD*, et al.* Monomeric red fluorescent proteins with a large Stokes shift. (2010).

13. Shcherbo D*, et al.* Near-infrared fluorescent proteins. *Nat Methods* **7**, 827-829 (2010).

14. Strack RL, Hein B, Bhattacharyya D, Hell SW, Keenan RJ, Glick BS. A rapidly maturing far-red derivative of DsRed-Express2 for whole-cell labeling. *Biochemistry* **48**, 8279-8281 (2009).

15. Bajar BT*, et al.* Fluorescent indicators for simultaneous reporting of all four cell cycle phases. *Nat Methods* **13**, 993-996 (2016).

16. Fabritius A, Ng D, Kist AM, Erdogan M, Portugues R, Griesbeck O. Imaging-Based Screening Platform Assists Protein Engineering. *Cell Chem Biol* **25**, 1554-1561.e1558 (2018).

17. Peterson J, Phillips GJ. New pSC101-derivative cloning vectors with elevated copy numbers. *Plasmid* **59**, 193-201 (2008).

18. Lutz R, Bujard H. Independent and tight regulation of transcriptional units in Escherichia coli via the LacR/O, the TetR/O and AraC/I1-I2 regulatory elements. *Nucleic Acids Res* **25**, 1203-1210 (1997).

19. Madeira F*, et al.* The EMBL-EBI search and sequence analysis tools APIs in 2019. *Nucleic Acids Res* **47**, W636-w641 (2019).
