# Supplementary material for: Orthogonal translation enables heterologous ribosome engineering in E. coli
Source: Nat Commun. 2021 Jan 26;12:599. doi: 10.1038/s41467-020-20759-z (PMC7838251; doi:10.1038/s41467-020-20759-z)
Supplement: Supplementary file 2 — Reporting Summary [file 41467_2020_20759_MOESM2_ESM.pdf]

nature research

Corresponding author(s): Ahmed H. Badran

Last updated by author(s): Dec 10, 2020

Reporting Summary

Nature Research wishes to improve the reproducibility of the work that we publish. This form provides structure for consistency and transparency in reporting. For further information on Nature Research policies, see Authors & Referees and the Editorial Policy Checklist .

Statistics

For all statistical analyses, confirm that the following items are present in the figure legend, table legend, main text, or Methods section.

n/a	Confirmed	
☐	☒	The exact sample size (n) for each experimental group/condition, given as a discrete number and unit of measurement
☐	☒	A statement on whether measurements were taken from distinct samples or whether the same sample was measured repeatedly
☒	☐	The statistical test(s) used AND whether they are one- or two-sided<br>*Only common tests should be described solely by name; describe more complex techniques in the Methods section.*
☒	☐	A description of all covariates tested
☒	☐	A description of any assumptions or corrections, such as tests of normality and adjustment for multiple comparisons
☐	☒	A full description of the statistical parameters including central tendency (e.g. means) or other basic estimates (e.g. regression coefficient) AND variation (e.g. standard deviation) or associated estimates of uncertainty (e.g. confidence intervals)
☒	☐	For null hypothesis testing, the test statistic (e.g. F, t, r) with confidence intervals, effect sizes, degrees of freedom and P value noted<br>*Give P values as exact values whenever suitable.*
☒	☐	For Bayesian analysis, information on the choice of priors and Markov chain Monte Carlo settings
☒	☐	For hierarchical and complex designs, identification of the appropriate level for tests and full reporting of outcomes
☒	☐	Estimates of effect sizes (e.g. Cohen's d, Pearson's r), indicating how they were calculated

Our web collection on statistics for biologists contains articles on many of the points above.

Software and code

Policy information about availability of computer code

Data collection	No software was used for data collection, except for software packaged with plate readers described in the manuscript (Tecan SparkControl v2.3 and Molecular Devices SoftMax Pro v6.4).
Data analysis	GraphPad Prism (version 8) was used for plotting and data analysis, including calculation of means, standard deviations, and regression lines. The Growthcurver package (version 0.3.0) in R (version 3.5.2) was used to calculate doubling times from growth curves.<br>Clustal Omega was used to generate alignments for sequence identities and to identify divergent 16S helices as described in the manuscript. NCBI BLAST (version 2.7.1) was used to calculate sequence similarity for r-proteins, as described in the Methods. The Ape package (version 5.3) in R (version 3.5.2) was used to compute distances for the phylogenetic tree in Figure 2d, as described in the Methods. The code used to do so can be accessed at https://github.com/sbratulic/HeterologousRibos. The tree was visualized using iTOL (version 5.7) (https://itol.embl.de/). RStudio (version 3.5.2) using the Biostrings (version 2.52.0) and TFBSTools (version 1.20.0) packages was used to calculate the conservation score in figure 3a, according to the equation in the citation (Valdar 2002).

For manuscripts utilizing custom algorithms or software that are central to the research but not yet described in published literature, software must be made available to editors/reviewers. We strongly encourage code deposition in a community repository (e.g. GitHub). See the Nature Research guidelines for submitting code & software for further information.

Data

Policy information about availability of data

All manuscripts must include a data availability statement. This statement should provide the following information, where applicable:
- Accession codes, unique identifiers, or web links for publicly available datasets
- A list of figures that have associated raw data
- A description of any restrictions on data availability

The authors declare that all the data supporting the findings of this study are available within the paper and its supplementary information files. The source data underlying Main Text Figures 1b-c, 2b-f, 3a, 3c-e, 4b-f, 5a-f, 6c-e, and Supplementary Figures 2a-h, 3b-p, 4, 5a-e, 6a-e, 7a-c, 9a-j, 10a, 10c-f, and 11a-n are provided as a Source Data File. The Genome Taxonomy Data Base phylogenetic tree used for phylogenetic analysis can be downloaded at https://data.ace.uq.edu.au/public/gtdb/data/releases/release86/86.1/. See Supplementary Table 6 for the correspondence between species names, NCBI taxIDs, NCBI species taxIDs, NCBI strain identifiers, and respective GTDB representative genomes. Key plasmids used in this study have been deposited in Addgene (see Supplementary Table 8 for Addgene IDs). All other relevant data are available from the authors upon reasonable request.

Field-specific reporting

Please select the one below that is the best fit for your research. If you are not sure, read the appropriate sections before making your selection.

☒ Life sciences ☐ Behavioural & social sciences ☐ Ecological, evolutionary & environmental sciences

For a reference copy of the document with all sections, see nature.com/documents/nr-reporting-summary-flat.pdf

Life sciences study design

All studies must disclose on these points even when the disclosure is negative.

Sample size	For some SQ171 growth assays, the minimal sample size (N=1) was determined by the number of colonies that passed Kan counter-screening against pSacB (as described in the Methods). Otherwise, the minimal sample size (generally N=8) was determined by the dimensions of a 96-well plate. Sample size is always reported in figure legends and/or in supplementary data tables.
Data exclusions	For SQ171 assays, colonies that did not pass Kan counter-screening against pSacB were excluded from analysis (as described in the Methods). Otherwise, no data were excluded.
Replication	For all experiments, the number of replicates is indicated in the figure legend.
Randomization	In this study, we systematically evaluated methods for improving the translational function of heterologous ribosomes in E. coli. All samples were evaluated using growth curves after SQ strain complementation or fluorescence measurements using orthogonal translation. No animals or humans were used in this study, so randomization was not necessary nor was it performed.
Blinding	No animal or human participants were used in this study, so blinding was not relevant.

Reporting for specific materials, systems and methods

We require information from authors about some types of materials, experimental systems and methods used in many studies. Here, indicate whether each material, system or method listed is relevant to your study. If you are not sure if a list item applies to your research, read the appropriate section before selecting a response.

Materials & experimental systems

n/a	Involved in the study	
☒	☐	Antibodies
☒	☐	Eukaryotic cell lines
☒	☐	Palaeontology
☒	☐	Animals and other organisms
☒	☐	Human research participants
☒	☐	Clinical data

Methods

n/a	Involved in the study	
☒	☐	ChIP-seq
☒	☐	Flow cytometry
☒	☐	MRI-based neuroimaging
